# Piezophototronic Effect in Nanosensors

**DOI:** 10.1002/smsc.202000060

**Published:** 2021-05-07

**Authors:** Rongrong Bao, Juan Tao, Caofeng Pan, Zhong Lin Wang

**Affiliations:** ^1^ CAS Center for Excellence in Nanoscience Beijing Key Laboratory of Micro-nano Energy and Sensor Beijing Institute of Nanoenergy and Nanosystems Chinese Academy of Sciences Beijing 100083 P. R. China; ^2^ School of Nanoscience and Technology University of Chinese Academy of Sciences Beijing 100049 P. R. China; ^3^ Center on Nanoenergy Research School of Physical Science and Technology Guangxi University Nanning Guangxi 530004 P. R. China; ^4^ College of Physics and Optoelectronic Engineering Shenzhen University Shenzhen 518060 P. R. China; ^5^ School of Materials Science and Engineering Georgia Institute of Technology Atlanta Georgia 30332-0245 USA

**Keywords:** nanosensors, optoelectronic devices, piezophototronic effects, pressure sensors

## Abstract

The piezophototronic effect is the coupling of piezoelectricity, semiconductor behavior and photon excitation in wurtzite materials, which can enhance the performance of optoelectronic nano‐devices by regulating a series of processes of carrier injection, transport, and recombination. In recent years, many research results have been reported on the improvement of nanophotoelectric nano‐device efficiency by piezophototronic effect, such as photovoltaic devices, light‐emitting devices, transistors, etc. Herein, the research results of piezophototronic effect in the field of nanosensors, including enhancing the performance of traditional nanosensors and new types of pressure/deformation/tactile sensors based on piezophototronic effect are comprehensively summarized. Finally, the future development of piezophototronic effect in the field of nanosensors is discussed.

## Graphical abstract

The research results of piezophototronic effect in the field of nanosensors, including enhancing the efficiency at semiconductor–metal contact or pn heterojunction by piezophototronic effect in traditional nanosensors and new types of pressure/deformation/tactile sensors based on piezophototronic effect are comprehensively reviewed. Also, the future development of piezophototronic effect in the field of nanosensors is discussed.

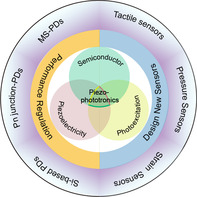

## 1 Introduction

The piezophototronic effect, which was introduced by Wang in 2010, is the coupling of piezoelectricity, semiconductor behavior, and photon excitation in wurtzite semiconductor materials, such as ZnO, GaN, and ZnS.^[^ ^1^, ^2^, ^3^, ^4^, ^5^, ^6^, ^7^ ^]^ When the optoelectronic nano‐devices are prepared from the piezophototronic nanomaterials under the strain, the piezo‐charges generated at both ends of the nanowires (NWs) form a piezopotential.^[^ ^8^, ^9^, ^10^, ^11^, ^12^ ^]^ The piezopotential can regulate the Schottky barrier height (SBH) at the interface of the electrodes and NWs or the energy level at the p–n junction, so that a series of processes of carrier injection, transport, and recombination will be controlled by the external stress. Therefore, piezophototronic effect is considered as an effective way to improve the efficiency of optoelectronic devices in addition to the structures of devices and characteristics of the materials.^[^ ^13^, ^14^, ^15^ ^]^ In recent years, many research results were reported on the improvement of nano photoelectric device performances by piezophototronic effect, such as nano photovoltaic devices,^[^ ^16^, ^17^, ^18^, ^19^, ^20^ ^]^ nano light‐emitting devices (LED),^[^ ^21^, ^22^, ^23^ ^]^ nano transistor devices,^[^ ^24^, ^25^, ^26^, ^27^ ^]^ etc. Among these photoelectric nano‐devices, sensors have become one of the research focuses because of their important role in intelligent electronics, information, Internet of Things, and other fields. The efficiency of traditional sensors such as photodetectors (PDs), humidity sensors, gas sensors, etc., can be improved by modulating the process of carrier transport through the piezophototronic effect at the surface region of junction under external stress.^[^ ^28^, ^29^, ^30^, ^31^ ^]^ On the other hand, the piezophototronic effect is also coupling among mechanical, optical, and electrical properties of photoelectric nano‐devices with the ability to regulate the carrier transport. Therefore, it provides a new way for the design and fabrication of pressure, strain, motion, and other new sensors.^[^ ^32^, ^33^, ^34^ ^]^ For example, the ultrahigh spatial resolution pressure‐distribution sensor based on NW LED array^[^ ^35^ ^]^ has been widely concerned by researchers for its first time to realize fast detecting beyond the spatial resolution of human skin.

**Figure 1:**
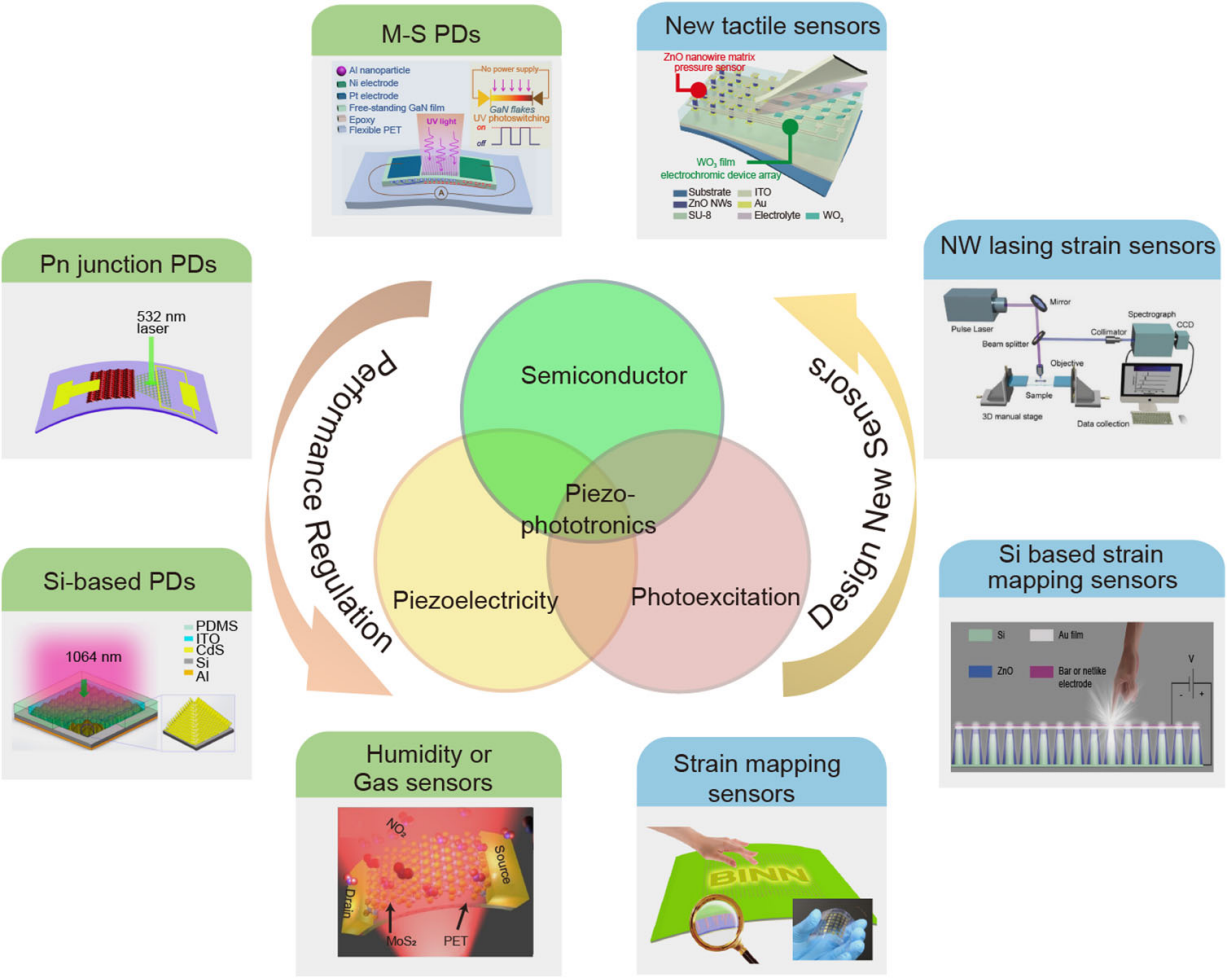
Two approaches to the application of piezophototronic effect in the field of nanosensors: enhancing the efficiency of traditional sensors or designing new types of high spatial resolution tactile sensors. “M‐S PDs” Reproduced with permission.^[^ ^39^ ^]^ Copyright 2015, American Chemical Society. “Pn junction PDs” Reproduced with permission.^[^ ^58^ ^]^ Copyright 2017, Royal Society of Chemistry. “Si‐based PDs” Reproduced with permission.^[^ ^60^ ^]^ Copyright 2017, American Chemical Society. “Humidity or Gas sensors” Reproduced with permission.^[^ ^66^ ^]^ Copyright 2019, Elsevier Ltd. “Strain mapping sensors” Reproduced with permission.^[^ ^84^ ^]^ Copyright 2015, Wiley‐VCH. “Si based strain mapping sensors” Reproduced with permission.^[^ ^88^ ^]^ Copyright 2015, Wiley‐VCH. “NW lasing strain sensors” Reproduced with permission.^[^ ^94^ ^]^ Copyright 2019, Wiley‐VCH. “New tactile sensors” Reproduced with permission.^[^ ^96^ ^]^ Copyright 2017, Wiley‐VCH.

In this review, we will comprehensively review the research results of piezophototronic effect in the field of nanosensors (**Figure** **1**). First, results of enhancing the efficiency by piezophototronic effect in PDs with the basic units of semiconductor–metal contact or p–n heterojunction will be systematically summarized. Second, we will introduce some new types of pressure/deformation/tactile sensors based on piezophototronic effect. Also their excellent performance compared with other pressure sensors and their potential applications in a wide range of fields is demonstrated. Finally, the flexoelectronic effect which is the latest research progress of piezophototronic effect has been briefly introduced and its application in the sensor design and fabrication is discussed.

## 2 Improvement in PD Performance by Piezophototronic Effect

Traditional nanosensors mainly include PDs, humidity/gas sensors, and so on. In recent years, many studies on the device structures and material characteristics have been carried out by scientists to improve the sensor performance. The research results of piezophototronic effect in the nanosensors, which can use the built‐in electric field to modulate the process of carrier generation, separation, transport, and recombination at the interface to achieve the sensitivity enhancing, contributed to the advancement in this field. In this section, we will introduce the typical research results on the performances of PDs regulated by piezophototronic effect.

A PD is one of the most important sensors with wide application in fields of Internet of Things, artificial intelligence, and health and medical. Therefore, the research on PDs attracts tremendous attention in recent years. The cores of the piezophototronic effect to enhance the performance of devices are using the piezopotential generated by strain near the interface of metal–semiconductor (M–S) Schottky contacts or p–n heterojunctions inside nano‐semiconductors to modulate the charge transport/separation/recombination process. We will introduce the results of the study about PDs with these two different interface contacts, including sensors based on traditional materials or new materials (2D materials and perovskite materials), regulated by piezophototronic effect.

### 2.1 The Piezophototronic Effect Enhanced Performance of PDs Based on M–S Structure

**Figure 2:**
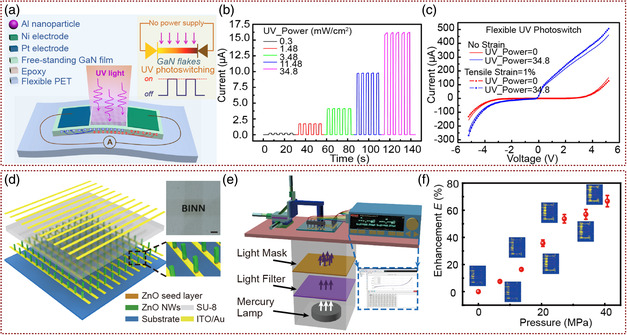
The piezophototronic effect enhanced PDs based on M–S structure. a) Schematic image of piezophototronic self‐powered GaN UV PDs based on MSM structure. b) The self‐powered on/off response of GaN UV PDs at 0 V bias. c) The photocurrent and dark‐current of the device were controlled to rise and decrease under stress, respectively. Therefore, the on/off ratio of the device was enhanced by the piezophototronic effect. a–c) Adapted with permission.^[^ ^39^ ^]^ Copyright 2015, American Chemical Society. d) Schematic illustration of piezophototronic ZnO NW UV‐PD array. e) Schematic of the measurement for piezophototronic effect enhanced ZnO NW UV‐PD array performance. f) The output current difference between ZnO NW arrays was used to map the distribution of UV illumination under the condition of strain free and strain (40.38 MPa), with 1.38 mW cm^−2^ illumination intensity and 1 V forward biased voltage. d–f) Adapted with permission.^[^ ^40^ ^]^ Copyright 2015, Wiley‐VCH.

The general and simple unit of piezophototronic PDs is the metal–semiconductor–metal (MSM) structure, which are usually fabricated by fixing two ends of NWs on a flexible polymer substrate with two back‐to‐back Schottky contacts at both ends of the semiconductor NWs. In 2010, the theory that the piezophototronic effect regulates the Schottky junction height of single ZnO NW MSM and thus affects the photocurrent was first reported by Yang, et al.^[^ ^36^ ^]^ They found that the Schottky barrier height (SBH) at the M–S interface is regulated by the excited light intensity and external pressure on the NWs at the same time. The SBH changes more at low light intensity than at high light intensity. The detection sensitivity can be enhanced by piezophototronic effect more than fivefold for pW levels of light detection. In 2012, the piezophototronic effect of CdSe NW–PDs with similar structures was reported by Pan and co‐workers, to show the flexible NW device sensitivity optimized by adjusting the applied strain.^[^ ^37^ ^]^ The piezophototronic effect under compressive strain adjusts the SBH near the interface of the M–S contact, which leads to the improvement in separation efficiency of photo‐excited electron–hole pairs and increase in photocurrent. They studied in detail that the photocurrent of the NW devices changed with the strain under different illumination intensity, and obtained the strain and light intensity conditions for the optimal device sensitivity. The piezophototronic effect was demonstrated to enhance the performance of devices constructed with not only traditional piezophototronic materials but also binary or ternary semiconductor nanostructures. Wang and co‐workers reported the piezophototronic effect on PDs of ternary CdS_*x*_Se_1−*x*_ NWs with different compositions.^[^ ^38^ ^]^ They studied and obtained the PDs with excellent photodetection ability, fast response, high photosensitivity, and photoresponsivity by piezophototronic effect modulation. The device efficiency can be regulated by both compositions and piezophototronic effects. The decrease in *x* in CdS_*x*_Se_1−*x*_ and the increase in tensile strain along the *c*‐axis direction of the NW reduce the SBH of M–S structure and induce more photogenerated carriers. Similar performance regulation was also reported in thin‐film PDs, such as the self‐powered UV PDs based on the GaN ﬂexible ﬁlm with high on/off ratio and excellent sensitivity, which was developed by Zhai and co‐workers (**Figure** **2**a,b).^[^ ^39^ ^]^ With the external stress, both built‐in electric field and piezoelectric polarization field drive the separation and transport of photogenerated carriers in the device. As its *I–V* characteristics shown in Figure 2c, the UV‐on current increased and the UV‐off current decreased by the strain‐modulation effect. Therefore, both the UV on/off ratio and sensitivity of the device were greatly improved, reaching to 4.67 × 10^5^ and 1.78 × 10^12^ cm Hz^0.5^ W^1−^, respectively. With 1% strain, the stronger and broader depletion region optimized device on/off ratio up to 154%. As the requirement of application, it is necessary to fabricate large‐area device arrays. Based on the study of the piezophototronic effect enhanced single NW PDs, Pan and co‐workers reported the UV PD array based on vertically aligned large‐area ZnO NWs, which consisted of 32 × 40 pixels.^[^ ^40^ ^]^ Schematic of the device structures is shown in Figure 2d. The top and bottom cross electrodes form a MSM with Schottky junction at both ends of each pixel in the array. Schematic of the measurement for ZnO NW UV–PD array performance enhancement by the piezophototronic effect is shown in Figure 2e. The optical responsivity, sensitivity, and detection limit of PD arrays were effectively increased by 700%, 600%, and 280% respectively, through strain‐induced piezoelectric polarization charges. By analyzing the output current difference between the pixels of UV PD array, it will be used for distributed imaging (Figure 2f) of UV illumination.

### 2.2 The Piezophototronic Effect Enhanced PDs Based on the P–N Junction

The sensitivity of p–n heterojunction PDs with the physical principle the same as the corresponding photovoltaic device, is improved by modulation of the energy band and barrier height through piezophototronic effect in the heterojunction region instead of SBH in MSM PDs. P–n heterojunction PDs have more device structures than MSM PDs, because of wide range of materials and structures to choose from. In this section, we will introduce piezophototronic p–n heterojunction PDs based on the core‐shell structures, vertical NW structures, and line‐film structures in turn, including the latest research on PDs based on 2D materials and perovskite materials.

**Figure 3:**
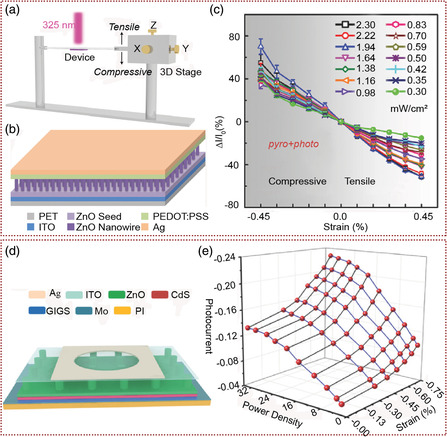
The piezophototronic effect enhanced p–n junction PDs based on core‐shell structure and vertical NW array structure. a) Schematic diagram of measurement for the PD performance enhancements by the piezophototronic effect. b) Schematic image of piezophototronic effect enhancing the performance of self‐powered organic/inorganic heterojunction PDs based on ZnO NWs/PEDOT:PSS layer. c) The photocurrent (*I*_pyro + photo_) under different strains and power densities. a–c) Adapted with permission.^[^ ^43^ ^]^ Copyright 2017, Wiley‐VCH. d) Structure diagram of the piezophototronic effect enhanced of the (CIGS) heterojunction PD. e) 3D graph depicting the CIGS heterojunction PD photocurrent under different compressive strains and laser power densities (−2 V forward biased voltage). d,e) Adapted with permission.^[^ ^44^ ^]^ Copyright 2018, Wiley‐VCH.

A piezophototronic effect enhanced flexible visible/UV PD based on ZnO/CdS core‐shell NW array was reported by Zhang, et al.^[^ ^41^ ^]^ CdS NW arrays were grown on the surface of ZnO NWs by hydrothermal method to form ZnO/CdS core‐shell nanostructures. Then, the two ends of this core‐shell NW were bonded on the flexible substrate to prepare a PD. Schematic diagram of measurement of the PD performance enhancement by the piezophototronic effect is shown in **Figure** **3**a. With the visible sensibility of CdS NW and the UV sensibility of ZnO NW, this device was sensitive simultaneously at two different excitation wavelengths (548 and 372 nm). When the device is under pressure, the strain‐induced piezopotential will increase the on/off ratio by modulation of the SBHs at the source and drain contacts. Similar to this device structure, the high‐performance UV/visible PDs fabricated on the ZnO/ZnS heterojunction core/shell NW array was reported by Zhou and co‐workers.^[^ ^42^ ^]^ By reducing the barrier height and modulating the carrier transport at the ZnO/ZnS interface by the piezophototronic effect, the detection sensitivities at three different excitation wavelengths (385, 465, and 520 nm) were all improved. This work showed a fabrication of broad band PDs based on core/shell NW arrays with the performance improved by the piezophototronic effect. Compared with the single NW PDs, the vertical NW arrays are more suitable for large‐area sensing, so it has been paid attention by researchers. For example, Wang and co‐workers designed and fabricated the flexible organic/inorganic heterojunction PD based on ZnO NW arrays and the PEDOT:PSS layer (Figure 3b).^[^ ^43^ ^]^ Then, the external strain was introduced to adjust the sensitivity of the device by combining pyrophototronic with piezophototronic effects. As shown in Figure 3c, the external strain can effectively regulate the separation and transport efficiency of photogenerated carriers and increase the photocurrent to more than 600%. In addition, the absolute photocurrent, rise time and fall time of PDs to 442 nm laser can be significantly improved using the pyroelectric effect induced by external compressive strain. Cu(In,Ga)Se_2_ (CIGS) has the advantages of strong light absorption, good stability, low production cost, and high efficiency and was considered to be a good optoelectronic device material. Pan and co‐workers have developed a flexible PD based on CIGS heterojunction.^[^ ^44^ ^]^ The structure of CIGS heterojunction PDs was polyimide (PI)/Mo/CIGS/CdS/ZnO NW array/Indium tin oxides (ITO) as shown in Figure 3d. The responsivity was 1.18 A W^−1^, the detection was 6.56 × 10^10^ Jones, and the response speed was about 70 ms. The performance of CIGS heterojunction was even better than that of traditional Si PDs. Moreover, the effective regulation of ZnO piezophototronic effect on carrier transport in heterostructures was also demonstrated. From the 3D graph depicting the photocurrent under different applied strains and light intensities shown in Figure 3e, we can see that the photocurrent increased significantly with the external pressure under different light intensities. The responsivity, detectivity, and response speed were increased by 75.4%, 66.1%, and 239.7%, respectively.

**Figure 4:**
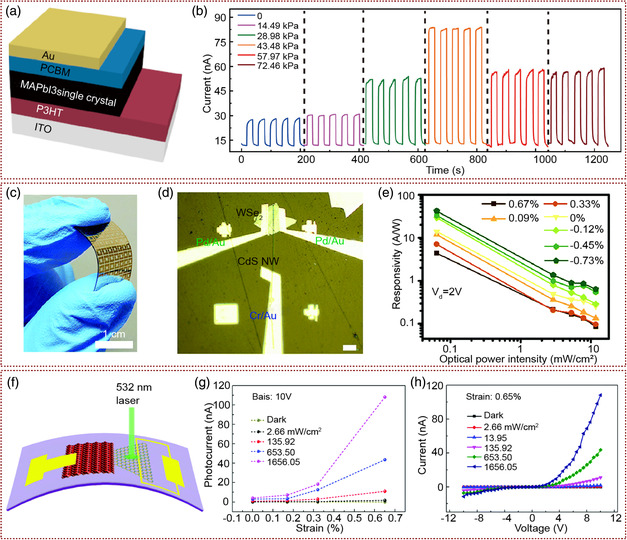
Piezophototronic effect improves the device efficiency of PDs based on perovskite materials or 2D materials. a) Device structure diagram of piezophototronic PD based on the MAPbI_3_ single crystal. b) *I–t* curves of the device under different pressures and 2.648 mW cm^−2^ power density at 1.5 V forward bias. a,b) Adapted with permission.^[^ ^54^ ^]^ Copyright 2018, American Chemical Society. c) The photo of a MAPbI_3_ single‐crystal PD being bent by fingers. d) The OM image of a MAPbI_3_ single‐crystal PD structure. e) Under externally applied stress, the responsivity of the WS_2_/CsPbBr_3_ PDs changes with different light intensity. c–e) Adapted with permission.^[^ ^56^ ^]^ Copyright 2019, Royal Society of Chemistry. f) The structure of single‐layer MoS_2_–CuO heterojunction PDs. g) Under different power densities, the photocurrent of the device increases with the stress. h) Under different power densities, the photocurrent of the device changes with the strain of 0.65%. f–h) Adapted with permission.^[^ ^58^ ^]^ Copyright 2017, Royal Society of Chemistry.

In recent years, new materials, such as 2D materials^[^ ^45^, ^46^, ^47^, ^48^ ^]^ and perovskite materials,^[^ ^49^, ^50^, ^51^, ^52^, ^53^ ^]^ have become one of research hotspots in design of photoelectric nano‐devices, because of their excellent photoelectric properties. Heterojunction PDs based on these materials with high performance, flexibility, and self‐powered can be developed by combining the piezophototronic effect and the advantages of 2D materials and perovskite materials. Organo lead‐halide perovskites with excellent carrier‐transport properties have developed rapidly in optoelectronic devices, especially in PDs. The way to enhance the performance of the perovskite‐based PDs has become one of the researchers focuses. Many effective methods, such as perovskite layer morphology improvement, have been reported. Among them, the performance of perovskite PDs regulated by piezophototronic effect was reported by Wang and co‐workers.^[^ ^54^ ^]^ They prepared MAPbI_3_ single crystals with much longer carrier‐diffusion length, low defect density, and a relatively large piezoelectric coefficient. Also fabricated the thin poly‐3‐hexylthiophene (P3HT) as hole transport layers and [6,6]‐phenyl‐C_61_‐butyric acid methyl ester (PCBM) as an electron transport layers above and below MAPbI_3_ single crystals to form the P3HT/MAPbI_3_/PCBM vertical structure. Au and ITO were used as upper and lower electrodes, respectively. The whole structure of the device is shown in **Figure** **4**a. As *I*−*t* curve of the device under different applied strain shown in Figure 4b, the performance of the device was greatly improved with the external pressure. Under 680 nm laser irradiation, the photocurrent of the PD was increased by 120% under the compression pressure of 43.48 kPa. This is because when the pressure is applied to the PD, the positive and negative electric charges are generated at the interface between the MAPbI_3_ single crystals and the electron/hole transport layer due to the piezophototronic effect, which facilitates the separation and reduces the recombination of photogenerated carriers. Furthermore, Nie et al. demonstrated a piezophototronic effect enhanced PD array based on polycrystalline perovskite.^[^ ^55^ ^]^ Compared with single‐crystal perovskite, polycrystalline perovskite materials are easier to prepare, lower environmental requirements, and higher conversion efficiency. Through systematic experiments, they studied the effects of excitation light, bias voltage, and different strain on the efficiency of polycrystalline perovskite PDs. The response speed of the device increased two to three times with the strain. Compared with single‐crystal perovskite devices, the research results of this work have much greater practicality. This work provided a new method for the preparation of high‐sensitivity polycrystalline perovskite PDs and modulation of device performance.

2D van der Waals (vdWs) heterostructures with possibilities to achieve precise control of the injection, separation, and recombination of carriers at the interface of heterojunction were reported as new PDs. A flexible 2D WSe_2_/1D CdS NW vdWs pn heterojunction PD^[^ ^56^ ^]^ was designed and fabricated and the device performance enhanced by piezophototronic effect was demonstrated by Wang and co‐workers. The photo of the device being bent by fingers and the optical microscopy (OM) image of device structure are shown in Figure 4c,d. They studied the optical responsivity of devices under different strains. The barrier height for holes was much larger than that for electron in the heterojunction. So, output current of the device was mainly determined by the electron diffusion current. The strain modulated the device performance through the piezoelectric polarization charge generated by the piezophototronic effect in CdS. It led to redistribute the carriers near the heterojunction contact, and then reduce the electron transport barrier and increase the photocurrent as nearly 110%. The optimum photo‐response can reach 33.4 under the −0.73% compressive strain (Figure 4e). With effective and ultrafast charge‐carrier transfer between perovskites and 2D materials, new planar heterojunction PDs were widely studied and reported. To avoid the low on/off ratio of planar 2D‐material/perovskite PDs, Pan and co‐workers determined a WS_2_/CsPbBr_3_ vdWH planar PD which consist of mechanically exfoliated 2D WS_2_ nanoflakes and single‐crystalline 1D CsPbBr_3_ NWs.^[^ ^57^ ^]^ The device has an on/off ratio of up to 10^9.83^, as well as strain gated and strain sensing due to the piezophototronic effect of CsPbBr_3_ NWs. Wang and co‐workers fabricated MoS_2_–CuO p–n heterojunction PD (Figure 4f) with single‐layer triangular MoS_2_ grown by chemical vapor deposition (CVD) method.^[^ ^58^ ^]^ As shown in Figure 4g,h, the *I–V* characteristics of MoS_2_–CuO heterojunction PDs under different light intensities and tensile stresses were measured. The results showed that the MoS_2_/CuO heterojunction has good p–n junction rectification characteristics. Under the same light intensity, the photocurrent increased greatly with the tensile stress on the positive bias voltage. Compared with the dark‐current, the photocurrent of the device was increased more clearly under stress. Furthermore, they studied the mechanism of piezophototronic effect to improve the light‐response performance of MoS_2_/CuO heterojunction PDs. Due to the noncentrosymmetric structure of the monolayer molybdenum disulfide, the positive and negative charge centers were separated under the tensile stress, and the positive polarization charge was induced at the MoS_2_/CuO heterojunction interface. The positive potential generated by this polarized charge intensified the bending of band structure on one side of MoS_2_, broadened the depletion region, and increased the built‐in electric field. Therefore, the recombination effect of the hole can be reduced and the optical performance of the device can be improved.

### 2.3 The Piezophototronic Effect Enhanced Near Infrared PDs Based on Silicon

In the design and fabrication of PDs, materials are often selected for detecting different wavelengths. Silicon (Si) with the absorption wavelength covers wide spectral range from UV to NIR, is one of the most important materials for PD applications. However, due to the low photon excitation energy and weak light absorption in the near‐infrared (NIR) band, the existing Si‐based PDs have a rapid decline in photoresponsivity, which limits the application to the visible spectrum. In third‐generation semiconductor materials (ZnO, CdS, GaN, etc.), the piezophototronic effect induced by mechanical strain can effectively regulate the photoelectric process of carriers, thus improving the device characteristics of PDs. Combining the advantages of Si and third‐generation semiconductor materials, we can fabricate the wide‐band heterojunction PDs with the performance improved by piezophototronic effect under external stress.

**Figure 5:**
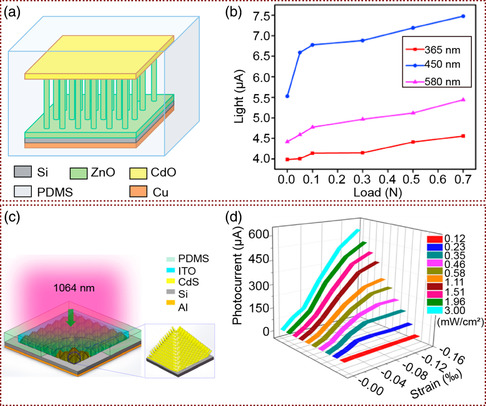
The piezophototronic effect enhanced NIR PDs based on Si. a) Schematic diagram of Si/ZnO/CdO three‐component heterojunctions PDs. b) The photocurrent with different pressure under the light stimulation of three wavelengths (365, 450, and 580 nm). a,b) Adapted with permission.^[^ ^59^ ^]^ Copyright 2017, Elsevier Ltd. c) Schematic diagram of NIR PDs based on n‐CdS NWs/textured‐Si substrate. d) 3D graph depicting the photocurrent under different strains and light power densities (forward bias is 2 V). c,d) Adapted with permission.^[^ ^60^ ^]^ Copyright 2017, American Chemical Society.

Hu and co‐workers reported a self‐powered and broadband optical‐response PDs based on Si/ZnO/CdO heterojunctions (**Figure** **5**a) with n‐ZnO NWs grown on p‐Si substrates by the hydrothermal process and CdO layer fabricated as the top electrode.^[^ ^59^ ^]^ The positive charges at the Si/ZnO interface and the negative charges at the ZnO/CdO interface generated by piezophototronic effect improved the photoresponse of PDs under compressive strain. As shown in Figure 5b, under the compression strain of 0.7 N, the maximum photocurrent under 365, 450, and 580 nm excitation light is increased by about 14.6%, 35.2%, and 23.2%, respectively. Dai et al. synthesized n‐CdS NWs by hydrothermal method on the textured‐Si substrate to fabricate p–n heterostructure NW NIR PD, which performance was enhanced by the piezophototronic effect (Figure 5c).^[^ ^60^ ^]^ Under 2 V forward bias voltage, when −0.15‰ external static compressive strain was applied, the optical responsivity R increased from 79.7 mA W^−1^ to 19.4 A W^−1^, which was about 366 times (Figure 5d). At the same time, the rise time and fall time of device decreased from 63 to 9.7 ms and 36 to 8.6 ms, respectively. Furthermore, they demonstrated that the performance of NIR PDs based on Si/CdS heterojunction with similar structures.^[^ ^61^ ^]^ Two heterojunction devices, based on p–n junction and n–n junction, were fabricated on p‐type silicon substrate and n‐type Si substrate respectively, and the influence of piezophototronic effect on device performance was studied. The enhancement of the p–n junction PD performance was much greater than that of n–n junction PD performance. For p–n junction PD, the optical responsivity increased by 966 times when the applied compressive strain was −0.50‰ and the response speed was increased at the same time, whereas for n–n junction PD, the optical responsivity only increased by six times under the same external compressive strain. The results were due to the different band structures of the two contacts of p–n and n–n junction.

### 2.4 Enhancement of Gas Sensors Performance by Piezophototronic Effect

**Figure 6:**
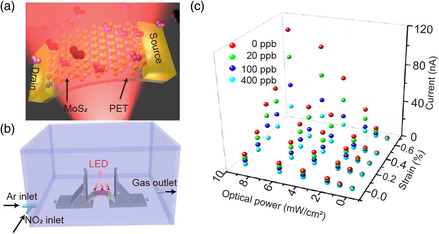
Enhancement of gas sensors performance by piezophototronic effect. a) Schematic illustration of enhanced detection of MoS_2_ NO_2_ sensors by piezophototronic effect on the flexible substrate. b) Schematic of the measurement for MoS_2_ NO_2_ sensors sensitivity. c) 3D graph depicting the current of the gas sensor under different applied strains, NO_2_ concentrations, and LED power densities. a–c) Adapted with permission.^[^ ^66^ ^]^ Copyright 2019, Elsevier Ltd.

In addition to PDs, piezophototronic effect can also improve the performance of other sensors, such as humidity sensors and gas sensors,^[^ ^62^, ^63^, ^64^, ^65^ ^]^ which are commonly used in our daily life, scientific research, industrial production, and other fields. SBH at M–S interface is regulated by both excitation intensity and external pressure. We can adjust the performance of these sensors by piezophototronic effect through by photogating, such as a flexible NO_2_ sensor based on single‐layer MoS_2_ reported by Wang and co‐workers.^[^ ^66^ ^]^ From the schematic illustration of enhanced detection of MoS_2_ sensors on the flexible substrate shown in **Figure** **6**a, we can see that, the monolayer MoS_2_ grown by carrying out CVD method was transferred onto flexible substrate, and then the electrodes were on both sides of monolayer MoS_2_ to obtain a flexible gas sensor. As shown in Figure 6b, the sensitivity of the device is measured in a sealed chamber under the illumination of red LED. Strain‐induced piezo‐charges can effectively control electron and photoelectron transport by modulating SBH. As can be seen from the 3D diagram shown in Figure 6c, when NO_2_ is 400 ppb (parts per billion), the sensitivity of the single‐layer sensor is increased to 671% under 0.67% tensile strain and 625 nm red LED illumination with 4 mW cm^−2^ power.

## 3 Ultrahigh Spatial Resolution Pressure Mapping Sensor Based on Piezophototronic Effect of the NW LEDs Array

In the previous sections, we introduced the research results of enhancing the sensor performance using the piezophototronic effect to regulate the carrier transport. With the development of artificial intelligence technology and robot science, more and more researchers pay attention to the large‐area tactile sensors with high spatial resolution. Most of the flexible large‐area tactile sensors made of elastic polymer or organic materials are based on the change of capacitance or resistance under the pressure.^[^ ^67^, ^68^, ^69^, ^70^, ^71^, ^72^, ^73^, ^74^, ^75^, ^76^ ^]^ The working principle of these devices makes it difficult to design sensor arrays with high spatial resolution and high integration, which greatly limits the application of these sensors. The piezophototronic effect can transfer mechanical energy into electrical and light energy, which provides a possibility for the development of new tactile sensors. With arrays of optoelectronic devices based on piezophototornic NWs, each nano‐device in arrays (usually in the nanometer or micron scale) is seem as a pixel to respond to the pressure changes.^[^ ^77^ ^]^ So, the spatial resolution of this type of devices is one order higher than that of human skin (about 50 μm). When the nano‐device array is under pressure, the piezophototronic effect regulates the carrier transport and recombination in the device, to change the device efficiency. The pressure distribution on the whole array can be obtained by analyzing the efficiency change of each device in the array. Recently, many novel tactile sensors based on piezophototronic effect were designed and studied, among which the pressure‐distribution sensor based on NW LEDs has been the most concerned. In this section, we will introduce the research on the enhancement of the performance and luminous intensity of NW LEDs by piezophototronic effect, and then introduce the design and preparation of high spatial resolution visualization pressure‐distribution sensors based on the piezophototronic NW LED arrays.

### 3.1 Brief Introduction of Piezophototronic Effect Enhanced the NW‐LED Performance

The piezophototronic effect is a new way to regulate the luminous intensity and efficiency of LED, which has been widely researched recently. A single NW‐LED based on piezoelectric semiconductor materials was first fabricated with the Mg‐doped p‐type GaN film and the n‐type ZnO NW by Wang and co‐workers in 2011.^[^ ^78^ ^]^ When the stress was applied above the device, the local biased voltage band modified caused by the piezopotential and the trapping of free carriers at the p–n junction. It led to the increase in carrier recombination at the p–n junction interface regions and enhancement of the device luminous efficiency and intensity. In 2014, Wang and co‐workers developed a methodology of 2D simulation for the piezophototronic effect on NW LEDs, and provided an optimization strategy for the future design of NW LEDs.^[^ ^79^ ^]^ Furthermore, similar performance‐enhanced processes have been also reported in a hybridized inorganic/organic NW LED with the structure of n‐ZnO NW/p‐polymer.^[^ ^80^, ^81^ ^]^ Under the external pressure, the electron mobility can be adjusted by the piezoelectric charges generated by the piezophototronic effect on the ZnO side to match the hole mobility near the p–n junction interface. Pan and co‐workers proved that the performance of n‐ZnO NWs grown vertically on p‐GaN substrate to form p–n junction LED can also be regulated by piezophototronic effect; the principle and technology of designing visual pressure‐distribution sensors based on NW LED arrays have been fully prepared. Furthermore, the flexible p–n junction LED arrays consisting n‐ZnO NWs and p‐polymer have also been designed and fabricated, which provided a feasible way for the fabrication of flexible visual pressure‐distribution sensors.

### 3.2 Ultrahigh Spatial Resolution Pressure Mapping Sensors Based on P–N Junction NW LED Arrays

**Figure 7:**
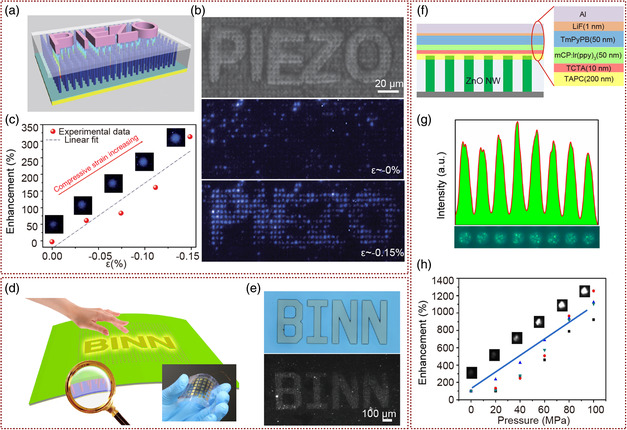
p–n junction NW LED pressure‐distribution sensors a) The working process of pressure‐distribution imaging based on GaN/ZnO heterojunction LED arrays. b) The curve of the luminous intensity of a LED‐pixel in the array increases linearly with the pressure. c) The optical of “PIEZO” convex sapphire used in pressure measurement and EL images of a device at strains of 0 and 0.15%. a–c) Adapted with permission.^[^ ^82^ ^]^ Copyright 2013, Springer Nature. d) Schematic illustration of the flexible ZnO/PEDOT:PSS heterojunction LED pressure‐distribution sensors. e) Optical image of LED array luminous intensity distribution change under pressure of the convex character pattern of “BINN”. d,e) Adapted with permission.^[^ ^84^ ^]^ Copyright 2015, Wiley‐VCH. f) Schematic of ZnO NW/OLED array. g) The OM image and corresponding emission intensity line profile of adjacent LED‐pixels in the array. h) The performance enhancement factor of a LED‐pixel in ZnO NW/OLED array increases linearly with the external pressure. f–h) Adapted with permission.^[^ ^87^ ^]^ Copyright 2017, American Chemical Society.

Pan and co‐workers have done a lot of excellent work and obtained exciting results in design and fabrication of pressure‐distribution sensors based on piezophototronic effect of NW LED arrays. They first designed a pressure‐distribution sensor consisting of n‐ZnO NWs grown on the p‐GaN layer to form a p–n junction LED array. The sensor was proved to be able to achieve a 2D strain distribution mapping with an unprecedented spatial resolution of 2.7 μm, corresponding to the pixel density of 6350 dpi as early as 2013 (**Figure** **7**a).^[^ ^82^ ^]^ As shown in the optical image of “PIEZO” convex sapphire used in pressure measurement and electroluminescence (EL) images of the device (Figure 7b) at strains of 0, and 0.15%, the brightness of the each LED‐pixel under pressure and deformation is enhanced, and there was little change in LED‐pixels without pressure. Under the compressive stress, the negative piezopotential generated by the piezophototronic effect improved the recombination rate of electrons and holes at the interface region of ZnO/GaN p–n junction, which led to enhancement of the EL intensity of NW‐LEDs at a near‐liner trend (Figure 7c). By taking photos continuously, the change in LED light intensity of each pixel in the array can be read in parallel, and the pressure distribution be monitored in real time. Compared with pressure‐distribution sensors by reading the electrical signals point by point, this NW LED arrays based sensor provided a new way to realize high spatial resolution sensing in a large area. Their research results achieved to obtain a visual pressure‐distribution sensor beyond the spatial resolution of human skin for the first time. However, epitaxial growth of GaN layer on sapphire substrate limits its application in the field of flexible sensing. To be used as an electronic skin, the flexible LED array based on the flexible GaN substrate or on the p‐polymer layer have been researched by Pan and co‐workers. On the one hand, they realized to directly grow ZnO NW arrays on the flexible GaN substrate through laser lift‐off (LLO) and transfer process to form a LED‐based pressure sensor.^[^ ^83^ ^]^ A copper film (about 50 μm thick) was prepared on sapphire/GaN substrate by electroplating. Then, GaN film was obtained by LLO and transferred to flexible substrate. Then, the flexible n‐ZnO/p‐GaN heterostructure LED arrays were fabricated by photolithography and hydrothermal method in the same process of preparing p–n junction LED arrays on hard sapphire/GaN substrates. When the pressure was applied, based on the piezophototronic effect, the region under pressure produces local compressive strain, which enhanced the luminescence of each LED‐pixel. Then, the pressure distribution was obtained by reading the light intensity of all LED‐pixels in parallel, which realized high spatial resolution of 2.6 μm and fast response time of 180 ms. This pressure sensor array with the advantages of flexibility, high resolution, fast response and transparency, will have application potential in the fields of intelligent skin, biomedicine, optical MEMS, and touch‐screen technology. On the other hand, flexible p‐type polymer materials replaced the rigid GaN layer and formed p–n junction LED arrays on flexible substrates with n‐type ZnO NWs.^[^ ^84^ ^]^ The device with a spatial resolution of 7 μm for mapping pressure was reported with the vertical growth PEDOT:PSS/n‐ZnO NWs p–n heterojunction LED array (Figure 7d). From the optical image of LED array luminous intensity distribution change under pressure of the convex character pattern of “BINN” as shown in Figure 7e, the flexible sensor achieved ultrahigh resolution pressure‐distribution sensing, similar to the hard devices based on sapphire/GaN substrate. Moreover, by controlling the growth conditions of ZnO NW array, the measuring range of pressure‐distribution sensors can be adjusted from 40–100 MPa. The similar LED array pressure sensors have also been studied in any other semiconductor materials and device structures. The PEDOT:PSS/n‐CdS junction LED array^[^ ^85^ ^]^ and the Au–SiO_2_–CdS metal–insulator–semiconductor (MIS) LED^[^ ^86^ ^]^ has been demonstrated by Pan and co‐workers. The EL intensity of the device depends on the electron–hole recombination inter CdS NW and which can be regulated by the piezophototronic effect. The spatial resolution of this device in pressure‐distribution mapping was as high as 1.5 μm, which is the highest value of pressure sensors been reported at present. In 2017, they combined the advantages of organic light‐emitting diodes (OLEDs) and piezophototronic effect of ZnO NW array to fabricate a color‐controllable high‐efficiency flexible pressure‐distribution sensor.^[^ ^87^ ^]^ As the structure of the device shown in Figure 7f, the OLED layer was prepared on the patterned ZnO NW array. From the OM image and corresponding emission intensity line profile of adjacent LED‐pixels in the array (Figure 7g), the spatial resolution of the array device was completely determined by the size and density of ZnO NWs, which was due to the carrier mobility of organic materials was much lower than that of inorganic materials in this device, whereas the color and intensity of luminescence were determined by OLED. So, the color of the device can be adjusted using different organic fluorescent materials in the light‐emitting layer. When the ZnO NW array is under pressure, the piezophototronic effect modulates the current of OLED, thus changing the luminous intensity. From the performance enhancement of one LED‐pixel in array increases linearly with the external pressure as shown in Figure 7h, the ZnO NW/OLED array will be used as a color‐adjustable ultrahigh resolution visual pressure‐distribution sensor.

### 3.3 Si‐Based Ultrahigh Spatial Resolution Pressure Mapping Sensors

Si with its excellent performance to ensure the rapid development of the Internet and personal computer, is earth‐abundant, cheap, and has a set of matching microelectronic technology, which is known as the most popular semiconductor material in the world. Si NW array has a very large specific surface area, excellent optical absorption and thermal conductivity, rich sources, low cost, and simple preparation. It will be used in many fields, such as field‐effect transistors (FETs), solar cells, biosensors, thermoelectric devices, and so on. However, Si is not suitable for fabrication of LEDs because of its indirect bandgap, which limits its development in the field of optoelectronic communication. At the same time, although all kinds of devices can be fabricated on silicon wafer, because Si is a very brittle material, Si‐based flexible devices have always been a great challenge.

**Figure 8:**
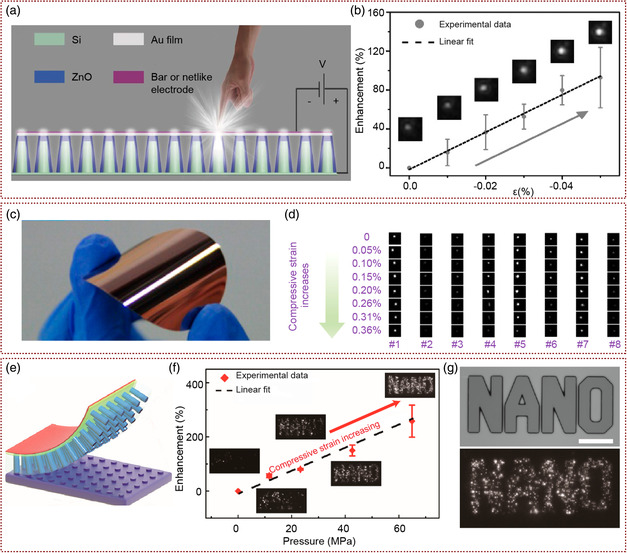
Si‐based NW LED pressure‐distribution sensors. a) Schematic image of ZnO‐nanofilm/Si‐micropillar heterostructure pressure sensors. b) Enhancement of LEDs luminous intensity under different strains. Insets are the corresponding light emission images of one LED‐pixel in the array. a,b) Adapted with permission.^[^ ^88^ ^]^ Copyright 2015, Wiley‐VCH. c) The optical image of a flexible SZHM device. d) The CCD images of single LEDs light intensity in array changes under increasing compressive strains. c,d) Adapted with permission.^[^ ^89^ ^]^ Copyright 2016, American Chemical Society. e) Schematic demonstration of the flexible NW array LED pressure‐distribution sensors based on the transferred Si microwires–ZnO nanofilm. f) Enhancement of LED luminous intensity under different pressure. g) The sapphire stamp with the word “NANO” pattern and corresponding photo of pressure‐distribution mapping by the LEDs array. e–g) Adapted with permission.^[^ ^90^ ^]^ Copyright 2017, American Chemical Society.

In recent years, Pan and co‐workers have done a lot of work on the fabrication of flexible Si‐based NW LED pressure‐distribution mapping sensors. They have not only realized Si‐based LEDs at room temperature but also demonstrated that the light‐emitting intensity of the devices can be modulated by the pressure. From the schematic demonstration and the SEM images shown in **Figure** **8**a, they reported p–n heterostructure LEDs consisting of Si wafers etched by inductively coupled plasma (ICP) and ZnO films to achieve light emissions at room temperature.^[^ ^88^ ^]^ From n‐type ZnO nanofilms and p‐type Si microcolumns heterojunction, this LED arrays emitted a wide range of white light from visible to NIR at room temperature from defects emissions in ZnO nanofilm, the energy bandgap of Si near the ZnO/Si p–n junction and Si microstructure. The intensity of this LED‐pixel in this device array was enhanced by 120% under −0.05% compressive strains (Figure 8b). This kind of high‐performance Si‐based LED array will have application potential in the field of electronic skin, and will be fully compatible with the dominant Si microelectronics industry. Then, they achieved to design and fabricate a similar structure of p‐Si/n‐ZnO heterojunction LED on a flexible Si substrate (Figure 8c).^[^ ^89^ ^]^ The device was based on the Si micropillar/ZnO heterostructure matrix (SZHM), which grows the ZnO NWs on the microstructured flexible silicon wafers through low‐temperature hydrothermal methods. Stress‐based light intensity control was achieved by piezophototronic effect in the p‐Si/n‐ZnO system. From the eight CCD images of different LED light intensity changes under increasing compressive strains, they found that the light‐emission intensity first increased and then decreased. Also the maximum emission intensity is obtained when compressive strain was 0.15–0.2% (Figure 8d). This was due to the positive piezoelectric charges produced by the piezophototronic effect, which created a dip in the local band structure near the interface of p–n junction, and regulated the electron transportation, recombination, and light emitting. When the conduction band edge of ZnO decreased to a level that the edges of both p‐side and n‐side were aligned, the emission intensity was improved to the highest. Furthermore, they had successfully fabricated flexible p‐Si/n‐ZnO LED arrays by embedding the etched Si NW arrays into the PDMS polymer layer and then peeled off the Si substrate (Figure 8e).^[^ ^90^ ^]^ During the transfer process, the orientation, structure, and etch pattern of the Si wire array were not changed, and the luminous intensity of flexible p‐Si/n‐ZnO LED array increased under pressure. The device still worked after 1000 bending times or storage in atmospheric for 6 months. As shown in Figure 8f,g, similar with other n‐ZnO/p‐Si heterojunction LED arrays, the intensity of the device was regulated by piezophototronic effect. Therefore, the device can also be used as a flexible pressure‐distribution mapping sensor, which has broad application in the fields of intelligent electronic skin, flexible touch‐screen, and electronic signature. The flexible Si‐based LED array device adopts a process compatible with the existing silicon microelectronics technology, which can directly use the existing production line to achieve large‐scale preparation, and provide strong support for the future integrated optical path.

## 4 Tactile Sensors Based on Piezophototronic Effect

Piezophototronic effect provides a way to convert mechanical energy into electrical energy by regulating the injection, transport, and recombination process of carriers in devices. Therefore, pressure sensors can be obtained by piezophototronic effect modulated by the efficiency of photoelectric devices. In recent years, with the research of single NW lasing, electrochromic device, power regulator and other optoelectronic devices, a variety of novel tactile sensors based on piezophototronic effect have been designed and fabricated by researchers.

### 4.1 Strain Sensors Based on Dynamic Regulating of Single‐NW Lasing by Piezophototronic Effect

**Figure 9:**
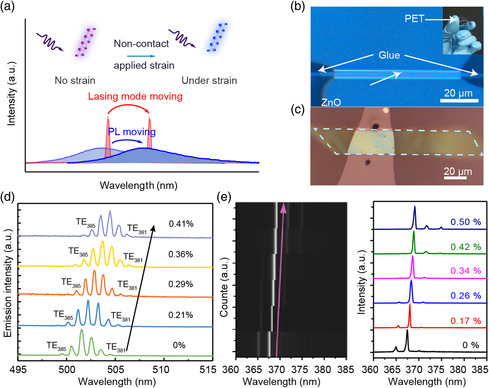
Strain sensor based on dynamic regulating of single‐NW lasing by piezophototronic effect. a) Schematic diagram of dynamic modulation of NW laser mode with and without strain. Adapted with permission.^[^ ^94^ ^]^ Copyright 2019, Wiley‐VCH. b) Optical image of the single ZnO NW with the two ends fixed on the flexible substrate. Adapted with permission.^[^ ^91^ ^]^ Copyright 2018, American Chemical Society. c) The optical image of a CdS parallelogram nanobelt with the two ends fixed on the flexible substrate. d) Strain dependence of the laser emission of the CdS nanobelt. c,d) Adapted with permission.^[^ ^93^ ^]^ Copyright 2019, American Chemical Society. e) Lasing spectra of GaN microcavity based strain sensor under different external tensile strain. Adapted with permission.^[^ ^94^ ^]^ Copyright 2019, Wiley‐VCH.

The continuous development of strain sensors provides an important opportunity for improving human–machine interface and health monitoring. To expand the application and prepare the strain sensor with higher performance, Pan and co‐workers studied dynamic modulating of the lasing spectra of semiconductor NWs by piezoelectric polarization and fabricated the strain sensors based on it (**Figure** **9**a). As shown in optical image in Figure 9b, they designed and fabricated a single ZnO NW‐based whispering‐gallery‐mode microcavity with both ends of the NW fixed on a flexible substrate.^[^ ^91^ ^]^ The refractive index of ZnO microcavity can be effectively controlled by applying stress on the flexible substrate, and the dynamic controlling of coherent lasing can be realized through the modulation of piezophototronic effect of wurtzite structure. By establishing the corresponding relationship between strain and lasing mode modulation, the super precision stress sensing was obtained. The lasing mode was distinguished clearly under a tensile strain of 0.51%. The research results provided an effective method for dynamic control of coherent light sources, and also a new way for color‐resolution stress sensors development. Reducing the cavity size is an effective way to control the mode and realize the single‐mode lasing. However, the realization of single‐mode lasing needs to reduce the microcavity size below the micron scale, which will seriously affect the output quality of lasers. Using the piezoresistive and piezoelectric polarization synergistic effect, the cavity size was maintained on the micron scale, and the dynamic regulating of single‐mode laser were achieved by Pan and co‐workers.^[^ ^92^ ^]^ At the same time, they also systematically analyzed the causes of single mode and provided a feasible scheme for further fabrication of color‐resolved stress sensors. To solve the problem that both piezoelectric polarization and resonant cavity length can control the laser mode simultaneously under strain, Pan and co‐workers used wurtzite‐structured CdS nanobelts as Fabry–Pérot (F–P) laser resonator, and investigate laser mode shift in different CdS crystal orientations under stress.^[^ ^93^ ^]^ Two morphologies of CdS nanobelts were prepared by VLS method. Among them, the growth direction of parallelogram nanobelts was along the [001] orientation (Figure 9c), whereas the growth direction of ladder nanobelts was non‐[001] orientation. The shift of laser modes induced by strain tuning corresponded to these two shapes and was closely related to the [001] orientation of CdS nanobelts. According to the F–P laser formula (Equation (1)), piezoelectric polarization leads to the effective refractive index (*n*_eff_) and the redshift of laser mode.

(1) $$\lambda =2n_{\text{eff}}L/N$$

In contrast, the cavity length (*L*) decreases and leads to the blue shift of lasing mode, due to the Poisson effect. As shown in Figure 9d, when the tensile strain was 0.41%, the red‐shift value of the laser reached 2.9 nm, which was almost twice the blue‐shift value (1.6 nm), indicating that the piezophototronic effect had a stronger regulation ability than the cavity length change. Therefore, in this study, the mechanism of strain‐controlling laser mode was clarified, and a new concept of noncontact stress sensing was proposed by combining the PL spectrum, Raman spectrum, and laser mode shift under stress. Furthermore, they transferred the GaN microwires synthesized by CVD method to the flexible substrates, and then studied the GaN microcavity lasing mode under strain by applying pressure on the substrate.^[^ ^94^ ^]^ Due to the piezoelectric polarization effect of wurtzite structure GaN, the piezophototronic effect will affect the refractive index of GaN, so as to achieve the dynamic controlling the laser mode under stress (Figure 9e). The refractive index of GaN changed linearly with the increase in applied tensile strain. Based on the piezophototronic effect of GaN, a flexible, high‐resolution, color‐adjustable, noncontact and simple structure strain sensor was designed by dynamically adjusting and controlling the laser mode of GaN. It will be used for noncontact stress measurement, remote strain detection, color‐adjustable pressure mapping, and optical signal modulation in the future extreme conditions.

### 4.2 Other New Types of Piezophototronic Pressure/Strain/Motion Sensors

**Figure 10:**
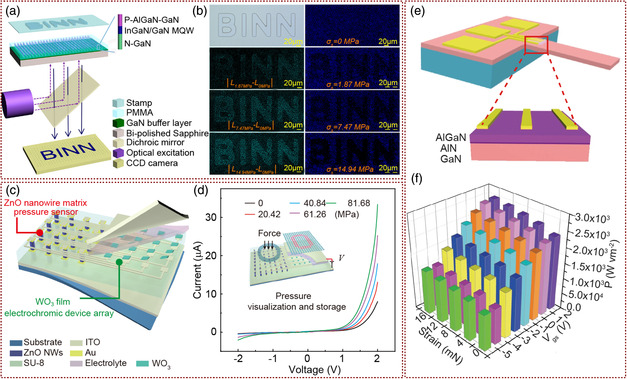
New types of pressure/strain/motion sensor based on piezophototronic effect. a) Schematic diagram of a PL pressure‐distribution sensor structure. b) Changes in PL intensity inside and outside the BINN stamp with different external compressive stress. a,b) Adapted with permission.^[^ ^95^ ^]^ Copyright 2015, American Chemical Society. c) Schematic illustration of the structure of the PVR system. d) Schematic illustration of piezophototronic effect working mechanism on PVR system. *I*–*V* characteristics of single ZnO NW pixel under different pressures. c,d) Adapted with permission.^[^ ^96^ ^]^ Copyright 2017, Wiley‐VCH. e) Schematic illustration of human spinal reflex like strain‐controlled power devices based on AlGaN/AlN/GaN‐microcantilever HEMT. f) 3D graph depicting the output power density of the strain‐controlled power devices under different strains and gate voltages. e,f) Reproduced under the terms of the CC‐BY 4.0 license.^[^ ^104^ ^]^ Copyright 2020, The Authors, published by Springer Nature.

Similar to the LED array pressure sensor, piezophototronic effect can also be used as the theoretical basis for the preparation of pressure sensors based on the photoluminescence NW array. Zhai and co‐workers reported a new dynamic pressure‐distribution mapping sensor based on photoluminescence imaging tuned by piezophototronic effect (**Figure** **10**a). The pressure‐distribution mapping sensor was based on vertical nanorod arrays which consisted of InGaN/GaN MQWs.^[^ ^95^ ^]^ The photoluminescence intensity of this device array was modulated by piezophtotronic effect linearly under strain (0–0.15%) (Figure 10b). In addition to real‐time mapping of plane pressure distribution, Pan and co‐workers developed a pressure visualization and recording (PVR) systems based on the WO_3_ film and ZnO NW arrays pressure sensor (Figure 10c).^[^ ^96^ ^]^ The whole pressure‐distribution visualization recording and storage system which achieves a spatial resolution of 500 μm. With the increasing stress on ZnO NW array, the current generated by piezophototronic effect of ZnO NW array enhanced, which affected the voltage applied on the WO_3_ film, and leads to the color changes of electrochromic layer and the direct visualization and storage of stress (Figure 10d). The coloration and bleaching process of ECD component was stability, and the color contrast was maintained above 85% after 300 cycles.

FET is an important device in the field of logic circuit design. When combined with FET, piezoelectric material which converts the external mechanical signals into electrical signals can realize the control performance of devices by external stress.^[^ ^97^, ^98^, ^99^, ^100^ ^]^ For example, Wang and co‐workers used piezoelectret MoS_2_ FET to achieve static/dynamic modulation.^[^ ^101^ ^]^ They used a piezoelectret material poly‐(vinylidenefluoride‐co‐trifluoroethylene) (P(VDF‐TrFE)) as the gate to realize the control of the single‐layer MoS_2_ FET under different polarization voltage and strain conditions, and obtained the stress sensor with high sensitivity, faster response time, and high stability. Compared with other sensors based on FETs, this sensor directly provided gate voltage under the action of stress, and no external power supply was required during the operation of the device. Therefore, it provided a new idea for the development of self‐powered devices in the future, and also the possibility of application in the following fields such as touch screen, artificial electronic skin, and so on. Using wet etching/transfer process, Hu and co‐workers achieved to fabricate a flexible large‐scale, wafer‐level AlGaN/GaN high electron mobility transistor (HEMT) arrays for the first time.^[^ ^102^ ^]^ The saturation current of the flexible HEMT array device had reached 290 mA mm^−1^. In recent years, more and more researchers have paid attention to new sensors imitating the human somatosensory system. For example, Sun and co‐workers reported a graphene artificial sensory synapse based on piezophototronic effect.^[^ ^103^ ^]^ The system included sensing, transmitting, and processing units. Based on the electric double layer formed at the interface of the ionic gel/graphene, the piezopotential effectively controlled the artificial synapses by piezophototronic effect. Wang and co‐workers designed and fabricated a sensor that can adjust the output power according to external stress by imitating the human spinal reflex.^[^ ^104^ ^]^ As shown in Figure 10e, the structure of the device was based on AlGaN/AlN/GaN‐microcantilever HEMT. Under the external force on the GaN‐microcantilever, the piezopotential generated by the piezophototronic effect modulated the output power of the HEMT. Different from the traditional tactile sensors with complex and low‐feedback speed signal‐processing systems, this device can obtain the external stress information in real time through the change in piezophototronic charges, and then quickly realize the feedback output power (Figure 10f). Meanwhile, the piezophototronic effect regulated the output power of HEMT through the piezopotential generated by the microcantilever, without disturbing the modulation of gate voltage on the output power of FET. Therefore, this power control device can be used in conjunction with other tactile sensing systems, just as the body's spinal reflex and the brain's control of muscle movements exist at the same time.

## 5 Conclusion and Perspective

In this article, we introduce the optimization of sensor performance by piezophototronic effect. It includes the modulation of the SBH of sensors by piezophototronic effect to obtain higher sensitivity, and using LED array based on piezophototronic effect to design new types of pressure sensors. Piezophototronic effect is the coupling among mechanical, optical, and electrical properties of semiconductor materials. It uses internal piezopotential to enhance the device performance instead of materials or device structures and has made great progress in the fields of intelligent sensing, human–machine interface, and robots, recently. However, piezophototronic effect only exists in noncentrosymmetric semiconductor materials, which greatly limits its application in the first generation of element semiconductors (such as Si) and the second generation of compound semiconductors (such as GaAs, GaP, and InP). Therefore, it is still a challenge to explore the introduction of similar interface regulation effects in semiconductors with centrosymmetric structures.

The flexoelectric effect is induced by strain gradient, which causes separation of positive and negative charge centers inter materials through nonuniform strain, and then generates polarization potential. It provides a way to realize piezophototronic effect like modulation in centrosymmetric semiconductor. Recently, Wang and co‐workers reported the flexoelectronic effect in bulk centrosymmetric semiconductors such as Si, TiO_2_, and Nb–SrTiO_3_ to achieve to obtain nanosensors with high strain sensitivity (>2650).^[^ ^105^ ^]^ The principle of flexoelectronic effect is similar to the piezophototronic effect, which uses the polarization potential near the metal–semiconductor interface as the gate voltage to effectively control the Schottky barrier at the interface, thus controlling the carrier transport characteristics at the interface. Different from piezophototronic effect, the flexure polarization caused by local nonuniform strain not only exists on the surface of the material but also distributes inside the materials over a range of lengths or volumes. Therefore, the flexoelectronic effect can be seemed as an integration of the piezophototronic effect over a length or volume or area. The studies of flexoelectric effect in semiconductors with centrosymmetric structures extended the research on piezophototronic effect from noncentrosymmetric third‐generation semiconductors to first‐ and second‐generation semiconductors, which is of great significance to the performance control of flexible sensors. For example, flexoelectric effect may improve the performance of existing Si‐based infrared detectors. It is similar to the regulation on the performance of ZnO PDs by piezophototronic effect introduced in the second section of this article. The flexoelectric effect of Si combined with nano‐OLED may obtain the ultrahigh spatial resolution pressure mapping sensors with adjustable luminous color. Compared with the nano‐LED array pressure sensors based on the third‐generation semiconductor materials, Si‐based pressure sensors will be more suitable for the existing chip manufacturing process. We can predict that more and more sensors with high performance and new functions based on centrosymmetric semiconductor materials will be designed and manufactured with further studies of the flexoelectric effect, which will play many important roles in all the fields of our daily life.

## Conflict of Interest

The authors declare no conflict of interest.
